# The new pLAI (*lux *regulon based auto-inducible) expression system for recombinant protein production in *Escherichia coli*

**DOI:** 10.1186/1475-2859-11-3

**Published:** 2012-01-05

**Authors:** Salvatore Nocadello, Erwin Frans Swennen

**Affiliations:** 1 Novartis Vaccines and Diagnostics srl, 53100 Siena, Italy

**Keywords:** autoinduction, recombinant protein production, Quorum Sensing

## Abstract

**Background:**

After many years of intensive research, it is generally assumed that no universal expression system can exist for high-level production of a given recombinant protein. Among the different expression systems, the inducible systems are the most popular for their tight regulation. However, induction is in many cases less favorable due to the high cost and/or toxicity of inducers, incompatibilities with industrial scale-up or detrimental growth conditions. Expression systems using autoinduction (or self-induction) prove to be extremely versatile allowing growth and induction of recombinant proteins without the need to monitor cell density or add inducer. Unfortunately, almost all the actual auto inducible expression systems need endogenous or induced metabolic changes during the growth to trigger induction, both frequently linked to detrimental condition to cell growth. In this context, we use a simple modular approach for a cell density-based genetic regulation in order to assemble an autoinducible recombinant protein expression system in *E. coli*.

**Result:**

The newly designed pLAI expression system places the expression of recombinant proteins in *Escherichia coli *under control of the regulatory genes of the *lux *regulon of *Vibrio fischeri*'s Quorum Sensing (QS) system.

The pLAI system allows a tight regulation of the recombinant gene allowing a negligible basal expression and expression only at high cell density. Sequence optimization of regulative genes of QS of *V. fischeri *for expression in *E. coli *upgraded the system to high level expression. Moreover, partition of regulative genes between the plasmid and the host genome and introduction of a molecular safety lock permitted tighter control of gene expression.

**Conclusion:**

Coupling gene expression to cell density using cell-to-cell communication provides a promising approach for recombinant protein production. The system allows the control of expression of the target recombinant gene independently from external inducers or drastic changes in metabolic conditions and enabling tight regulation of expression.

## Background

After many years of intensive research on expression of heterologous proteins, some empirical "rules" guiding the design of expression systems have emerged. Among these, tight regulation of promoter activity allows a fast initial period of cell growth to high density. Once an optimal cell density is obtained, protein expression can be triggered through inducible activation of the promoter. The promoters P*_lac_*, P*_trp_*, P*_tac_*, λP*_L_*, P_T*7*_, P*_BAD_*, P_*lacUV*5 _and P_T*5 *_are commonly utilized in this approach [1-3].

However, despite the availability of excellent expression systems for high-level production of recombinant proteins, the approaches using inducible promoters require monitoring of the metabolic state and cell density. In addition, balancing the cell growth rate with the rate of heterologous protein production is required to maximize the overall levels of protein production [4,5]. The different inducible expression systems offer few possibilities to fine-tune gene expression and a heterogeneous uptake of the inducer by the cells of the culture is very common [6-10]. Finally, the high cost and potential toxicity of inducers, such as isopropyl-b-D-thiogalactoside (IPTG), limit their use for industrial scale protein expression and for production of therapeutic proteins [11-20].

The use of auto-inducing (or self-inducing) expression systems for recombinant protein production eliminates the necessity to monitor cell growth and to actively induce the expression of a target gene at the appropriate moment and to the desired level. Unfortunately, almost all auto-inducible promoters respond to the metabolic state of population or to the imposition of specific culture conditions. Furthermore, several reported auto-induction mechanisms are regulated by growth rate and a number of intracellular metabolic signals as well as specific growth conditions, such as catabolite repression, nutrient availability, dissolved oxygen tension, pH and osmolarity. These auto-induction approaches demand reactions that could be detrimental or unnecessary for production purposes [21-25].

Among known regulatory systems, Quorum Sensing (QS) offers a unique opportunity to link the expression of a recombinant gene to population density and enable auto-induction under fine regulated genetic control. Recently, Tsao et al. [26] re-engineered the *native *QS regulon of *Escherichia coli *to initiate and drive autonomously recombinant protein expression in response to prevailing metabolic state of the bacteria population. The circuit was designed to enabled modulated expression of the target gene under the control of P_t7 _promoter and the incorporated of a T7Rpol under the control of QS in a two-plasmid based expression system.

In the present study, we use a *heterologous *QS genetic circuit for recombinant protein expression in *E. coli *in order to provide a compact genetically engineered gene regulatory network that responds to cell density and that is minimally affected by the metabolic state of the host. The *Vibrio fischeri*'s genetic circuit for QS has been shown previously to work both in prokaryotic and eukaryotic cells in various innovative synthetic-biology applications [27-30]. We employ a simple modular design strategy to create a recombinant protein expression system in *E. coli *strains where a genetic 'toggle switch' is interfaced with the transgenic QS signaling pathway from *V. fischeri*. The LuxI protein, from the well-characterized LuxI/LuxR system, synthesizes a small and specific signal molecule N-3-(oxohexanoyl)-l-homoserine lactone (the auto-inducer, AI) [31,32]. LuxI produces the AI from a common precursor of the host's metabolism, S-adenosylmethionine, and an acylated acyl carrier protein from the fatty acid biosynthesis pathway [33]. As the cell density increases AI accumulates in the medium and inside the cells. At a critical concentration, the chemical equilibrium shifts favoring formation of a complex with the transcriptional factor LuxR. The AI-LuxR complex acts on a 20-bp palindromic sequence (the LUX BOX) and directs the over-expression of the P*_luxI _*promoter and the operon containing the gene. The diffusible nature of AI is the regulative key of the genetic circuit. It enables cell density-dependent induction of gene expression that is homogeneous in all cells of the culture [32]. Moreover, the genetic organization of *V. fischeri*'s Lux regulon was conserved in such a way that a positive feedback loop creates a very sensitive switch to turn on the expression without the necessity to amplify the signal with other complex genetic circuits [31].

## Results and discussion

### Construction and characteristics of expression vectors

**Figure 1:**
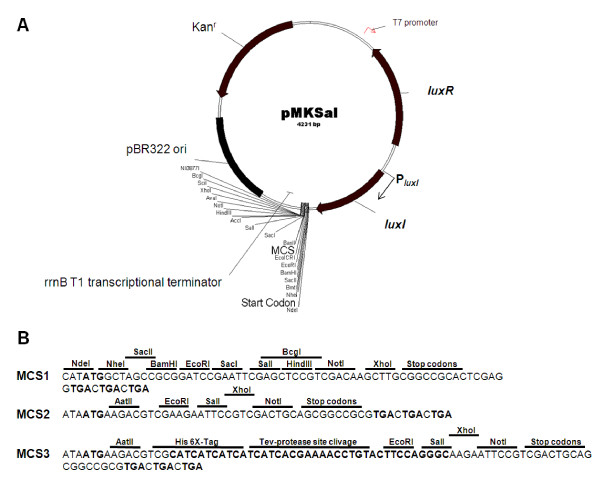
**Features of Vectors**. (A) Map of pMKSal as a representative of the pLAI vectors. (B) pMKSal and pMKSal-Δ*luxI *have the MCS1. MCS2 belongs to pLAIET32 and pLAIR32; MCS3 belongs to pLAIR42. Abbreviations: *rrnBT1 *part of the strong ribosomal *rrnB *terminator; ori, origin of replication; MCS, multiple cloning site.

The pLAI system is based on the *lux*I promoter, *P_luxI_*, the transcriptional activator gene, *luxR*, the *LUX-BOX*, and the *luxI *gene (Figure 1). However, additional features were included in the design of the pLAI expression system in order to build a set of expression vectors for a wider panel of options. His-tag, TEV protease cleavage site, MCSs, stop codons and *rrnb T1 *transcriptional terminator where considered and arranged on different vectors (Figure 1B). We designed a MCS compatible with other commercial vectors, simplifying cloning operations. In fact, MCS1 has compatible restriction sites with some MCS of vectors of the pET system (pET21 series). Moreover, the vectors contain a pBR322 origin of replication (*ori*) or a pUC *ori *and an antibiotic resistance gene (ampicillin (Ap^r^) or kanamycin (Km^r^)). The modular approach allows the incorporation of all regulatory elements in one plasmid (pMKSal, pLAIET32, pLAIR32 or pLAIR42) or the partition of regulative genes between the plasmid (pMKSal-*ΔluxI*) and the host genome (MM294.1::*luxI*). Furthermore, codon-optimized *luxI *and *luxR *and the IPTG inducible promoter P_T7 _were included in the pMKSal series. Promoter P_T7 _in divergent arrangement with respect to *luxR *was used in order to test the repression of QS by using inducible transcriptional interference, a molecular switch controlling repression of QS at high cell density.

### Optimization of the vector pMKSal

One of the most important properties of an expression system is the high rate of recombinant gene transcription that can be achieved. Induction of the *P_luxI _*promoter increases exponentially the strength of expression to approximately 10^7^-fold over the basal level [19]. These characteristics allow the protein to accumulate to very high concentration after induction, while deleterious effects of production of toxic gene products leading to growth inhibition should be minimized by maintaining the level of basal expression extremely low during the growth phase.

It is interesting to note that we found many rare codons in *luxI *and *luxR *for their translation in *E. coli*. In fact, ORFs of *luxI *and *luxR *have 16 and 11 codons, respectively, with an usage frequency lower than 30%. In order to allow efficient translation according to the codon usage of the host organism, the codon usage and the GC content of the gene sequences for AI-synthase *luxI *and the AI-dependent transcriptional factor *luxR*, were changed. The optimized *luxI *and *luxR *sequences have several codon modifications and are 74.742% and 74.235% identical to the original sequences, respectively. The GC% of *luxI *and *luxR *changed from 32% and 30%, respectively, to 46%. Afterwards, the use of pBR322 origin of replication for a medium plasmid copy number and a different antibiotic resistance cassette was evaluated in combination with the use of optimized *luxI *and *luxR *sequences in the pMKSal.

**Figure 2:**
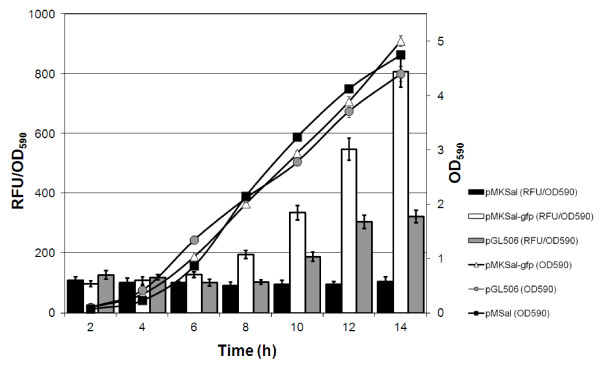
**Optimization of pMKSal vector**. Comparison of growth curve and culture-average fluorescence (Relative Fluorescence Unit per OD_590 _unit) of *E. coli *harboring pMKSal-*gfp *(*luxI *and *luxR *optimized; open triangles and white bar blots for OD_590 _and fluorescence, respectively), pGL506 (*luxI *and *luxR *non-optimized; grey circles and grey histogram for OD_590 _and fluorescence, respectively) and pMKSal (negative control, *luxI *and *luxR *optimized; black squares and black histogram for OD_590 _and fluorescence, respectively). Cell cultures were grown overnight in LB at 37°C and sub-cultured in CM medium with the appropriate antibiotic, to a final density of 0.15 OD_590_. 5 replicates of each clone were incubated in 96-well plates at 30°C with shaking, monitoring the optical density and the fluorescence in a Tecan Infinite M200. Error bars show the standard deviation of the experiment.

The optimization of pMKSal vector increases the expression of the reporter gene when compared with non optimized pGL506 vector (Figure 2). In fact, the fluorescence intensity of GFP increased to 250% in fully-induced conditions. The vector optimization also affected the threshold of response to cell density. In fact, the pMKSal-*gfp *harboring cells showed induction of expression at lower cell density than the vector containing the non-optimized control genes. At low cell density, the fluorescence value is not different from the negative control for both pMKsal-*gfp *and pGL506.

The optimization of pMKSal vector demonstrated to improve the expression level. The rare codons in the *V. fischeri*'s regulatory genes could affect their expression in *E. coli *and consequently the availability of LuxR and LuxI reducing the formation of the LuxR-AI complex. Furthermore, the sequence of *luxI *is particularly important considering the polycistronic expression of the recombinant gene and the mRNA stability that is associated with codon frequency [34]. Codon optimization should affect also the translation of *luxI *mRNA allowing the critical concentration of AI to be reached at lower cell density and triggering induction (Figure 2). Therefore, the optimization of pMKSal vector strongly affects the dynamic response of induction to cell density.

### Reduction of gene dosage of *luxI*

**Figure 3:**
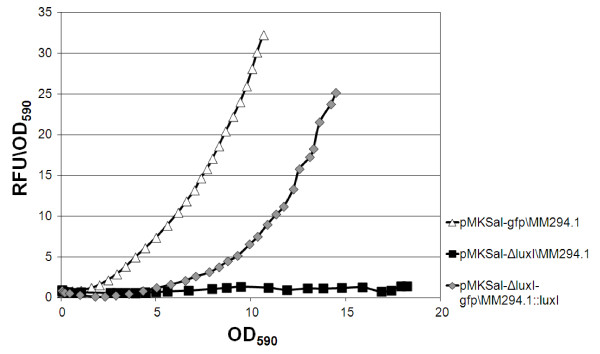
**Dynamic expression of the decoupled *luxI/luxR *auto-inducible system**. Comparison of culture-average fluorescence (Relative Fluorescence Unit per OD_590 _unit) and cell density associated with specific fluorescence of the decoupled *luxI/luxR *auto-inducible system (pMKSal-Δ*luxI-gfp*/MM294.1::*luxI*; grey diamonds), coupled *luxI/luxR *optimized-sequences auto-inducible system (pMKSal-*gfp*/MM294.1; open triangles), and control (pMKSal/MM294.1; solid squares). Cells carrying the plasmid, grown overnight in LB at 37°C, were sub-cultured in CM medium with the appropriate antibiotic, to a final density of 0.15 OD_590_. 5 replicates of each clone were incubated in 96-well plates at 27°C with shaking, in a Tecan Infinite M200, monitoring the optical density and the fluorescence. The temperature was decreased at 27°C in order to reach high cell density in rich medium with a low aerated condition.

Inducible expression systems fulfill a fundamental requirement regarding recombinant protein production compared to constitutive promoter-based systems with respect to their ability to uncouple biomass production from the expression of the target gene [35]. The rate of synthesis of AI determines the cell density at which the AI reaches the critical concentration to induce the expression. In order to obtain induction at higher cell density, we argue to reduce the rate of synthesis of AI, by reducing the rate of expression of AI synthetase. Therefore, the gene dosage of *luxI *was reduced by integrating non-optimized *luxI *gene into the genome of *E. coli *MM294.1 (see Materials and Methods) (see Additional file 1, Figure S1). The *luxI *gene was truncated in the plasmid pMKSal by using two sites for the *KpnI *restriction endonuclease that eliminates approximately 70% of the ORF to generate pMKSal-Δ*luxI *(see Additional file 1, Figure S2). Cell density induction, dynamic expression and growth rate of the decoupled *luxI/luxR *auto-inducible system (pMKSal-Δ*luxI-gfp*/MM294.1::*luxI) *was compared with plasmids carrying *luxI-luxR *optimized sequences (pMKSal*-gfp*) (Figure 3). As expected, the reduction of gene dosage of *luxI *in the pMKSal-Δ*luxI-gfp*/MM294.1::*luxI *combination resulted in induction of the *gfp *reporter gene at higher cell density. Although the specific fluorescence in fully induced conditions of the pMKSal-Δ*luxI-gfp*/MM294.1::*luxI *combination is reduced by about 20% with respect to pMKSal*-gfp*/MM294.1, induction at higher cell density resulted in a lower impact on the growth rate. A higher final cell density was obtained increasing the volumetric productivity (amount of product produced per liter) of approximately 10% measured by fluorescence.

Therefore, by reducing the gene dosage of *luxI *to 1 copy per genome the expression system is induced after reaching a higher cell density but with a stronger feedback effect than the other constructs used for comparison in this study. The data suggest that the uncoupling of the *luxI*-gene dosage from the plasmid-copy-number should leads to a tighter control of basal expression at low cell density. This strategy could be considered for production of toxic proteins.

### Repression of the auto-inducible system

A useful property of a tightly regulated expression system is the control of the un-induced state at high cell density. This feature is exploited during pre-culturing for the normalization of different growths using the "grown to saturation" strategy [25], or during scale-up in industrial processes.

In order to provide the genetic regulatory circuit with a system for repression a novel approach was developed. The molecular mechanism of repression is based on transcriptional interference as the suppressive influence of one transcriptional process, directly and *in cis *on a second transcriptional process [36,37].

To test the efficiency of repression of QS activation by the transcriptional interference mechanism, an inducible promoter, *P_T7_*, was introduced in divergent arrangement with respect to *luxR *in the pMKSal plasmid (Figure 1). *P_T7 _*promoter is induced on pMKSal plasmid only in the BL21(DE3) strain by IPTG. Furthermore, LuxR is required for cell density-dependent induction [38].

**Figure 4:**
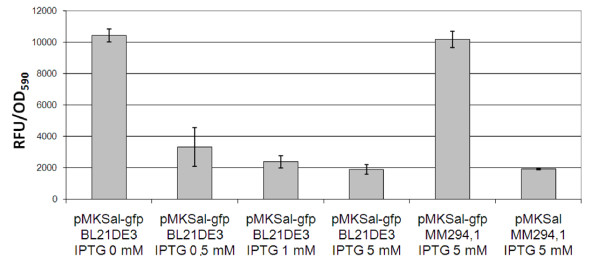
**Repression of the auto-inducuble system**. Comparison of efficiency of repression of the auto-inducible system by transcriptional interference on *luxR*. pMKSal-*gpf */MM294.1, pMKSal-*gpf */BL21DE3 and pMKSal/MM294.1 were grown in LB at 37°C with shaking. For each plasmid/strain combination, 3 replicates were incubated for each of the 4 concentrations of IPTG: 0, 0.5, 1 and 5 mM. After 16 h of growth, GFP expression was determined. pMKSal-*gpf */MM294.1 and pMKSal/MM294.1 combinations were used as positive and negative controls. Error bars show the standard deviation of experiments. RFU/OD_590_, Relative Fluorescence Unit per OD_590 _unit.

The fluorescence of pMKSal-*gfp*/BL21(DE3) without and with IPTG at different concentrations was compared in Figure 4. The presence of 1 mM of IPTG in the growth medium allowed complete repression of the *lux *regulatory circuit and the specific fluorescence was at comparable levels to the negative control (pMKSal/MM294.1).

Insertion of the transcriptional interference mechanism provides a useful approach to repress the expression of target genes. This property may be of advantage in industrial fermentation during the scale up process. The IPTG inducible promoter P_T7 _was used to test the repression of QS by using inducible transcriptional interference. However, the use of other inducible promoters could be explored

## Conclusion

Since Grossman et al. [39] encountered unintended induction in the T7 based expression system auto-induction of expression for recombinant protein production is generally referred to as a phenomenon intimately related to the medium composition. It allows induction and production of substantial amounts of target protein in absence of an added inducer. Consequently, many types of auto-inducible media were developed and auto-induction has become a standard procedure in many laboratories for testing expression and solubility of many proteins and for producing target proteins in large amounts for purification [25]. In fact, auto-induction is more convenient than inducer-dependent induction. The expression strain is inoculated into well-defined auto-inducing medium and grown to saturation without the need to monitor culture growth or to add an inducer at a specific point in time of the process. Any promoter that is induced by a change in metabolic state of the growth culture, such as the transition to depletion of a specific nutrient, catabolite repression, stationary growth, pH, oxygen levels or osmolarity, can be applied in the auto-inductive approach [22]. Nonetheless, most of these systems are strictly related to a particular medium definition and/or growth conditions, often causing stress upon the cells during growth and during the recombinant protein production phase.

This work describes a different approach for the auto-induction of recombinant protein expression. QS provides a strictly regulated genetic control enabling recombinant gene expression to be linked to population density. While Tsao et al. [27] developed a autoinducible system by rewiring native QS of *E. coli*, in our approach, we were looking for a simple molecular switch that was i) directly linked to cell-density, ii) independent from any endogenous signals and from reorganization of host's metabolic state. We demonstrated that the regulation of *V. fischeri*'s QS system, optimized in the pLAI expression system, is a very useful mechanism to control gene expression for protein production. Since *E. coli *is the prokaryotic expression host of choice for the production of many recombinant proteins, we adapted the simplified cell-density dependent genetic switch of *V. fischeri*'s QS to *E. coli *strains.

The amphipathic, diffusible character of AI simplifies the genetic regulatory circuit avoiding the necessity of a transporter for the inducer. In several available *E. coli *expression systems only all-or-none gene expression is possible because expression of the gene encoding the transporter for the inducer is controlled by the inducer itself [40-43]. In these expression systems, expression is not adjustable in individual cells and protein expression is not homogeneous. In contrast, the pLAI expression system ensures homogeneous induction due to the high permeability of the cells for AI [32].

The system does not need specific auto-inducible medium and/or growth conditions to guarantee auto-induction. Rather, the system responds directly to cell density during growth. However, growth conditions exist that allow modification of the cell density perception and thus also the protein expression profile. For example, it is reported that the half-life of AI affects cell-to-cell communication and the detection of cell density [38]. Indeed, the AI degradation rate is accelerated by increasing the pH of the medium [19]. Furthermore, the regulation of the LuxI/LuxR system of *V. fischeri *is also controlled by catabolite repression [44]. Our preliminary data suggest that pH and glucose concentration are useful fermentative variables that could be used to adjust the cell-density dependent induction or the dynamic expression pattern to respond to specific production requirements (see Additional file 1, Figures S3, S4, S5). However, more experimental data are needed.

In conclusion, we have generated an effective, autonomous system of auto-induction which does not rely on the addition of an inducer, the use of specific culture conditions or imposition of specific stress factors. Furthermore, it enables tight regulation of gene expression, homogeneous induction and high maximal expression. Different genetic approaches provided a panel of vectors that make the pLAI system very flexible for different protein production requirements.

Auto-induction is convenient, efficient and economical for production of proteins for research and industrial purposes since cultures for auto-induction are simply inoculated and grown to saturation. While direct application of this expression system in industrial scale is ongoing, we welcome these cell density-based autoinducible expression systems for high throughput recombinant protein expression and lab-scale production processes.

## Methods

### Media and bacterial strains

Cultures were grown in Luria-Bertani (LB) broth at 37°C or as indicated in the figure legends. Complex medium (CM) contains yeast extract (30 g/l), KH_2_PO_4 _5,4 g/l, K_2_HPO_4 _15,6 g/l, MgSO4 250 mg/l, glycerol 15 g/l, pH 7.4. YE3X medium contains yeast extract (45 g/l), KH_2_PO_4 _4 g/l, K_2_HPO_4 _16 g/l, glycerol 15 g/l, pH 7.2. Antibiotics were used as follows: ampicillin, 100 μg/ml; chloramphenicol, 30 μg/ml; kanamycin, 50 μg/ml and erythromycin 50 μg/ml. Media were supplemented with glucose or IPTG as indicated in the figure legends (the materials were obtained from Sigma, St. Louis, Mo.). Cell growth was monitored as the optical density at a wavelength of 590 nm (OD_590_).

The bacterial strains used in the present study are listed in Table 1. All DNA manipulations were performed in *E. coli *DH5α by using established protocols [23].

**Table 1:** Strains or plasmids used in this work.

Strains or plasmids	Descriptions^a^	References or sources
**Strains**		
***V. fischeri ***	*ATCC7744*	*ATCC7744*
***E. coli *DH5α**	*F' Φ80lacZΔM15 Δ(lacZYA-argF)U169 deoR recA1 endA1 hsdR17(r-k, m+k) phoA supE44 thi-1 gyrA96 relA1 λ^- ^*	*Invitrogen*
***E. coli *TOP10**	*F' mcrA Δ(mrr-hsdRMS-mcrBC) Φ80lacZ ΔM15 ΔlacΧ74 recA1 araD139 Δ(ara-leu) 7697 galU galK rpsL (Str^r^) endA1 nupG *	*Invitrogen*
***E. coli *MM294.1**	*F' supE44 hsdR17 endA1 thi-1 λ^-^*	*Novartis Collection*
***E. coli *MM294.1::luxI**	*F' supE44 hsdR17 endA1 thi-1 λ^- ^metE::ery\luxI*.	*This study*
***E. coli *BL21(DE3)**	*F' ompT gal dcm lon hsdSB(rB- mB-) λ(DE3 [lacI lacUV5-T7 gene 1 ind1 sam7 nin5])*	*Novagen*
**Plasmids**		
**pBS-Ery**	*pBR322 ori; Ap^r^; carrying ery (Er^r^);*	*51*
**pET21b**	*IPTG inducible; pBR322 ori; Ap^r^; *	*Novagen*
**pET21b-*gfp***	*pET21b carrying gfp; Ap^r^; *	*This study*
**pGlow**	*TOPO-TA vector; promoterless gfp; pUC ori; Ap^r^; *	*Invitrogen*
**pGL506**	*pGlow carrying luxR-luxI amplicon in TA cloning site; pUC ori; Ap^r^;*	*This study*
**pGLEM**	*pGlow carrying ΔmetE-ery in TA cloning site; pUC ori; Ap^r^;*	*Novartis Collection*
**pGLEM-*luxI***	*pGLEM carrying luxI; pUC ori; Ap^r^;*	*This study*
**pKobeg**	*Arabinose inducible expression for gam, bet, exo; R101 ori; Cm^r^;*	*7*
**pMK**	*pBR322 ori; Km^r^;*	*1*
**pMKSal**	*MCS1; pBR322 ori; Km^r^;*	*This study*
**pMKSal-GFP**	*pMKSal carrying gfp; Km^r^;*	*This study*
**pMKSal-*ΔluxI***	*pMKSal with truncated luxI; Km^r^;*	*This study*
**pMKSal-*ΔluxI*-GFP**	*pMKSal-ΔluxI carrying gfp; Km^r^;*	*This study*
**pLAIR32**	*MCS2; pUC ori; Ap^r ^and Km^r^;*	*This study*
**pLAIR42**	*MCS3; pUC ori; Ap^r ^and Km^r^;*	*This study*
**pLAIET32**	*MCS2; pBR322 ori; Ap^r^;*	*This study*

*^a ^*Ap^r^, ampicillin resistance; Cm^r^, chloramphenicol resistance; Km^r^, kanamycin resistance; Er^r^, Erythromycin resistance; *ori*, replication origin; IPTG, Isopropyl-β-D-thiogalactoside.

Strain *E. coli *MM294.1::*luxI *was constructed applying a modified PCR-mediated gene recombination method [45]. Briefly, *luxI *gene, including its promoter, was amplified by PCR using the primers LxI*AscI*F-TTTTGGCGCGCCCATTATTTCCCCTATAATATACTTAGTA and LxI*AscI*R-TTTTGGCGCGCCTAAAACGGTAATAGATTGACA and chromosomal DNA of *V. fischeri ATCC7744*. The PCR product was digested with *AscI*. Concomitantly, for pGLEM contructions, *metE *gene was amplified from genomic DNA of *E. coli *MM294.1 with the primers metE*ScaI*L-GAAAAGTACTGCTTGTAGCGTTTTCAGGTG and metE*ScaI*R- GAAAAGTACTGGGAAGAAGTCGCTGTAATG and cloned in pGLOW (Invitrogen). The restriction site for *EcoRV *in *metE *was used to produce blunt-ends to be ligated with a blunt-end fragment containing the *ery *gene, extracted from pBS-Ery with *SmaI *[46]. The restriction site for AscI in *ΔmetE*, proximal to the *ery *gene, was used to insert the digested PCR product containing non codon-optimized *luxI*, generating pGLEM-*luxI*. The entire linear fragment containing *ΔmetE-ery-luxI-ΔmetE *was PCR amplified from pGLEM-*luxI*, using MetEL-GAAAAGTACTGCTTGTAGCGTTTTCAGGTG and MetER-GAAAAGTACTGGGAAGAAGTCGCTGTAATG. The amplicon was used to transform *E.coli *MM294.1 carrying the pKobeg plasmid and erythromycin-resistant candidate integrants were analyzed by PCR (using LuxI4Fr-TCAAATGTCAATCTATTACCG and LuxI4Rv-TCCTTACCTATTGTTTGTCG) and Southern blot [45].

Briefly, for Southern blot, genomic DNA was prepared from an overnight liquid culture of *E. coli *MM294.1 and its isogenic *luxI *mutant using the NucleoSpin Tissue kit (Macherey-Nagel GmbH & Co. KG, Düren, Germany). Five μg of genomic DNA of each mutant was digested overnight with *XmaI *and *AatII *restriction enzymes at 37°C and loaded on a 0.7% agarose gel with appropriate DNA size markers. A 513-bp DNA probe, annealing within the *luxI *gene, was prepared by PCR from pGLEM-*luxI *using the primers LuxI4Fr and LuxI4Rv. Southern blot was performed with the ECL Direct Nucleic Acid Labeling and Detection Systems kit (GE Healthcare) as described by the manufacturer.

### Construction of pLAI vectors

Constructed vectors differ in multicloning sites (MCS), the antibiotic resistant gene and origin of replication (*ori*).

The *luxR-luxI *containing region of the *lux *regulon was amplified from genomic DNA of *V. fisch*eri ATCC7744 with LuxIRFw-AAGCTTTACTTACGTACTTAACTTTTA and LuxIRRv-TCATTATTTCCCCTATAATATACTTAGT. The PCR product was inserted in topoisomerase recombination sites of pGLOW (Invitrogen) to produce pGL506. *luxR *and *luxI *genes were codon optimized as previously described by Richardson et al. [47]. Two enzymatic restriction sites, for *KpnI *and *XbaI*, were introduced in *luxI*. A DNA fragment containing the T7 promoter, the optimized *luxR *gene, the *luxR-luxI *intergenic region, the optimized *luxI *gene, a multiple cloning site (MCS1) and the transcription terminator was designed and synthesized (GeneArt). This fragment was inserted into a pMK vector using *AscI *and *PacI *cloning sites, producing pMKSal [48]. The *gfp *gene was amplified from pGLOW (Invitrogen) using GFPEco-GAATTCAATGGCTAGCAAAGGAGAAGAACT and GFPNotI-GCGGCCGCTTATTTGTAGAGCTCATCCA and inserted in the MCS1 of pMKSal using *EcoRI *and *NotI*. The final plasmid is pKMSal-*gfp*.

pMKSal-*ΔluxI *is a pMKSal derivative plasmid obtained by its digestion with *KpnI *and self-circularization in order to generate a non functional LuxI. For pLAIET32, pLAIR32 and pLAIR42 vector constructions, 2 DNA fragments, were assembled by using long primer and assembly-PCR, as previously reported [49]. Both sequences containing *MluI*-*AatII*-MCS2-*NdeI *or *MluI*-*AatII*-MCS3-*NdeI*, were cloned in pCRII (Invitrogen). The *luxR-luxI *containing fragment, amplified from pGL506 plasmid with LF*MluI*T-TTTTACGCGTTACTTACGTACTTAACTTTTA and LR*AatII*T-TTTTGACGTCTTCATTATTTCCCCTATAATATA, was inserted in sequences containing MCS2 or MCS3 on pCRII by using *MluI*/*AatII*, to generate pLAIR32 and pLAIR42. The sequence containing *luxR-luxI*-MCS2 was extracted from pLAIR32 by *MluI/NdeI *digestion. The PCR-product of pET21 (Novagen), pETORIAMP*MluI*Fr-TTTTACGCGTGAGAAGCAGGCCATTATCGC and pETORIAMP*NdeI*Rv-TTTTCATATGATTTCAGGTGGCACTTTTCG, containing pBR322 *or*i and the *bla *gene, was digested with *MluI/NdeI *and was ligated with the *luxR-luxI*-MCS2 DNA fragment. The final plasmid is pLAIET32. The enzymes were obtained from New England Biolabs.

### Transcriptional fusion studies of pGL506, pMKSal, pET21 promoters linked to the *gfp *reporter gene

A seed culture was made by inoculating cells into LB medium containing the appropriate antibiotic and growing the cells overnight at 37°C. Each seed culture was inoculated in fresh LB, YE3X or CM medium with antibiotics, to obtain a final optical density of 0.15 (OD_590_). The cells were grown at different temperatures, as indicated in figure legends. When the OD_590 _reached 0.5, the cells harboring the IPTG inducible promoter were induced with 1 mM of IPTG or as indicated in figure legends. GFP was used to provide an indirect, quantitative measurement of the transcriptional properties of the cloned gene [50,51].

GFP fluorescence in batch cultures of *E. coli *containing the reporter plasmids expressing *gfp *was measured by Tecan Infinite M200 plate reader (Tecan) using an excitation wavelength of 405 nm and an emission wavelength of 535 nm. GFP fluorescence was normalized for cell density (GFP fluorescence per OD_590 _unit).

### Protein extracts, SDS-polyacrylamide gel electrophoresis, and Western blotting

All samples for SDS-page or Western blotting were normalized by cell density. Cell cultures were centrifugated (13000 rpm for 5 min) and the pellets were resuspended in 1 ml of buffer B-PER (Thermo scientific), 10 μl of lysozyme (100 mg/ml, Invitrogen) and 20 μl of DNase (1000 kunits/ml, Invitrogen). The samples were incubated at room temperature for 30 min. To 120 μl of sample, 40 μl of Loading Sample Buffer 4 × (0.4 M DTT, 8% SDS, 200 mM Tris-HCl pH 6.8, 0.1% bromophenol blue, 40% glycerol) was added. The samples were boiled for 10 min at 95°C. For each sample, protein extracts were loaded on SDS-12% polyacrylamide gels, and polyacrylamide gel electrophoresis was performed at 180 mA for 1 h. Immunoblotting was performed with a commercial anti-GFP antibody (Invitrogen) as first primary antibody, as previously described [23].

## List of abbreviations

QS: Quorum Sensing; AI: autoinducer.

## Competing interests

Patent Application WO/2010/136897

## Authors' contributions

SN carried out the studies and drafted the manuscript. ES participated in the design of the study and coordination and revised the manuscript. All authors read and approved the final manuscript.

## Authors' information

SN, PhD, current position: post-doc at Novartis Vaccines and Diagnostics

ES, PhD, current position: lab head at Novartis Vaccines and Diagnostics.

## Supplementary Material

Additional file 1 **Figure S1. Molecular characterization of luxI integration into the E. coli genome**. A) PCR analysis of MM294.1::luxI. Chromosomal DNA from wild type MM294.1 (line 1), plasmid pGL506 (line 2) and chromosomal DNA of MM294.1::luxI (line 3) were used as a template for PCRs with primers LuxI4Fr/LuxI4Rv, specifically annealing to *luxI*. A molecular weight ladder is also shown, on the right. *B) *Southern blot analysis of wild type and MM294.1::luxI mutant strain. Plasmid pGLEM-luxI (line 3; positive control), Chromosomal DNA from MM294.1::luxI mutant (line 4) and wild type MM294.1 (line 5) were digested with XmaI\AatII restriction enzymes. Fragments were separated on an agarose gel and transferred to nitrocellulose membrane for Southern blot analysis using a 513-bp PCR (primers LuxI4Fr/LuxI4Rv) that fully probed within theluxI gene. As a control of the Southern blot efficiency, a 513-bp PCR (primers LuxI4Fr/LuxI4Rv) from plasmid pGL506 (line 1) and chromosomal DNA of MM294.1::luxI (line 2) were loaded onto the agarose gel. C) Schematic representation of *luxI i*ntegration into the genome. **Figure S2**. **Schematic representation of decoupled luxI/luxR auto-inducible system**. LuxI is produced from the genome, reducing the luxI gene dosage to 1/genome, while luxR and the target gene dosages depend on plasmid copy number of pMKSal-ΔluxI. **Figure S3**. **pH effect on the pattern of expression**. Comparison of pMKSal-gfp/MM294.1 expression at different cell densities at two different pH set points (6.2 ± 0.1 and 7.2 ± 0.1) controlled during bacterial growth in a bioreactor. Batch processes were carried out in a 7-liter bioreactor (Applikon) under the following conditions: 5L of YE3X containing 15 g/l of glycerol, 25°C, 0.5 VVM (*air Volume *per *Volume *of culture *medium *per Minute) of airflow and a 300-rpm stirrer speed. The glucose was added to the medium where indicated. An Applikon programmable logic controller (ADI1030) was used for maintaining temperature at 25°C and pH set-point. The pH of the medium was maintained with 2 M H_3_PO_4 _and 4 M NaOH. The concentration of dissolved oxygen was maintained at 65% by controlling the stirrer speed up to a maximum speed of 800 rpm and O_2 _flow by electronic valve. The sampling was done for SDS-PAGE, Western blotting and fluorescence assays as reported above. During the growth at 25°C samples were collected at the indicated cell density. The fluorescence level was determined for 3 replicates of each sample. Controls pMKSal/MM294.1 (negative) and pET21-gfp/BL21(DE3) (positive) were growth in the same conditions. pET21-gfp/BL21(DE3) was induced at 3OD590 with 1 mM of IPTG and collected after 3 hours from the induction. Controls pMKSal/MM294.1 and pET21-gfp/BL21(DE3) were collected at 13.4 OD_590 _and 12.5 OD_590 _respectively. Error bars show the standard deviation of the experiment. **Figure S4**. **Glucose effect on the pattern of expression**. Comparison of culture-average fluorescence (fluorescence per OD_590 _unit) of E. coli harboring pMKSal-gfp grown in LB (squares) or YE3X (triangles) without (open symbols) or with the addition of 2.5 g per liter of glucose (solid symbols). Cells grown overnight at 37°C in LB medium containing the appropriate antibiotic, were used to inoculate LB and YE3X media with antibiotic and with or without glucose, in 96-well plates at 27°C with shaking in a Tecan Infinite M200, monitoring the optical density and the fluorescence. 5 replicates of each condition were incubated. **Figure S5**. **Western blot analysis of GFP expression during the batch process under conditions of catabolite repression**. E. coli cells harboring pGL506 grown overnight at 37°C in shaking a flask with YE3X with the appropriate antibiotic were used to inoculate 7 L Applikon bioreactor containing 5 L of YE3X medium with glucose (1 g/L), 15 g/l of glycerol. The process was performed at 25°C, with 0.5 VVM (*air Volume *per *Volume *of culture *medium *per Minute) of airflow and a 300-rpm stirrer speed. An Applikon programmable logic controller (ADI1030) was used for maintaining temperature at 25°C and pH set-point. The pH of the medium was maintained with 2 M H_3_PO_4 _and 4 M NaOH. The concentration of dissolved oxygen was maintained at 65% by controlling the stirrer speed up to a maximum speed of 800 rpm and O_2 _flow by electronic valve. Samples were collected at the indicated cell densities. Total proteins were isolated at various times at the cell density reported in each well. The proteins were separated on an SDS 10% polyacrylamide gel and blotted onto a nitrocellulose membrane to be incubated with an anti-GFP polyclonal antibody (Invitrogen). All samples were normalized based on the biomass. As a positive control, 0.2 μg of purified GFP (Invitrogen) was used. Click here for file

## References

[B1] Baneyx F Recombinant protein expression in *Escherichia coli* Curr Opin Biotechnol 1999 10 411 421 10.1016/s0958-1669(99)00003-8 10508629

[B2] Choi YJ Morel L Le François T Bourque D Bourget L Groleau D Massie B Míguez CB Novel, versatile, and tightly regulated expression system for *Escherichia coli *strains Appl Environ Microbiol 2010 76 5058 5066 10.1128/AEM.00413-10 PMC2916507 20562288

[B3] Hanahan D Glover DM Techniques for transformation of *Escherichia coli*. DNA cloning: a practical approach 1985 109 135

[B4] Guda C Zhang X McPherson DT Xu J Cherry JH Urry DW Daniell H Hyper expression of an environmentally friendly synthetic polymer gene Biotechnology Letters 1995 17 745 750

[B5] Masui Y Mizuno T Nouye M Novel high-level expression cloning vehicles: 10^4^-fold amplification of *Escherichia coli *minor protein Bio-Technology 1984 2 81 85

[B6] Giacalone MJ Toxic protein expression in *Escherichia coli *using a rhamnose-based tightly regulated and tunable promoter system Biotechniques 2006 40 596 596 10.2144/000112112 16568824

[B7] Kurland CG Dong HJ Bacterial growth inhibition by overproduction of protein Molecular Microbiology 1996 21 1 4 10.1046/j.1365-2958.1996.5901313.x 8843428

[B8] Patkar A Vijayasankaran N Urry DW Srienc F Flow cytometry as a useful tool for process development: rapid evaluation of expression systems Journal of Biotechnology 2002 93 217 229 10.1016/s0168-1656(01)00399-6 11755986

[B9] Schein CH Noteborn MHM Formation of soluble recombinant proteins in *Escherichia coli *is favored by lower growth temperature Bio-Technology 1988 6 291 294

[B10] Sevastsyanovich YR Alfasi SN Cole JA Sense and nonsense from a systems biology approach to microbial recombinant protein production Biotechnology and Applied Biochemistry 2010 55 9 28 10.1042/BA20090174 20044926

[B11] Andrews B Adari H Hanning G Lahue E Gosselin M Martin S Ahmed A Ford PJ Hayman EG Makrides SC A tightly regulated high level expression vector that utilizes a thermosensitive lac repressor: production of the human T cell receptor V beta 5.3 in *Escherichia coli* Gene 1996 182 101 10.1016/s0378-1119(96)00523-9 8982074

[B12] Bhandari P Gowrishankar J An *Escherichia coli *host strain useful for efficient overproduction of cloned gene products with NaCl as the inducer J Bacterio 1997 179 4403 4406 10.1128/jb.179.13.4403-4406.1997 PMC179267 9209061

[B13] Darby RAJ Hine AV LacI-mediated sequence-specific affinity purification of plasmid DNA for therapeutic applications Faseb Journal 2005 19 801 10.1096/fj.04-2812fje 15760969

[B14] Figge J Wright C Collins CJ Roberts TM Livingston DM Stringent regulation of stably integrated chloranphenicol acetyl transferase genes by *Escherichia coli *Lac repressor in monkey cells Cell 1988 52 713 722 10.1016/0092-8674(88)90409-6 2830990

[B15] Jana S Deb JK Strategies for efficient production of heterologous proteins in *Escherichia coli* Applied Microbiology and Biotechnology 2005 67 289 298 10.1007/s00253-004-1814-0 15635462

[B16] Knodler LA Sekyere EO Stewart TS Schofield PJ Edwards MR Cloning and expression of a prokaryotic enzyme, arginine deiminase, from a primitive eukaryote *Giardia intestinalis* Journal of Biological Chemistry 1998 273 4470 4477 10.1074/jbc.273.8.4470 9468500

[B17] Kosinski MJ Rinas U Bailey JE Isopropyl-β-D-thiogtalactopyranoside onfluences the metabolism of Escherichia coli Applied Microbiology and Biotechnology 1992 36 782 784

[B18] Pal Y Gupta JC Mukherjee KJ Optimizing recombinant protein expression in the T7 system under the control of the *proUp *promoter Biotechnology Letters 2001 23 41 46

[B19] Thomas MD van Tilburg A Overexpression of foreign proteins using the *Vibrio fischeri lux *control system Bioluminescence and Chemiluminescence, Pt C 2000 305 315 329 10.1016/s0076-6879(00)05497-5 10812610

[B20] Schumann W Production of recombinant proteins in *Bacillus subtilis* Advances in Applied Microbiology, Vol 62 2007 62 137 189 10.1016/S0065-2164(07)62006-1 17869605

[B21] Lee SK Keasling JD A propionate-inducible expression system for enteric bacteria Applied and Environmental Microbiology 2005 71 6856 6862 10.1128/AEM.71.11.6856-6862.2005 PMC1287719 16269719

[B22] Miksch G Bettenworth F Friehs K Flaschel E Saalbach A Twellmann T Nattkemper TW Libraries of synthetic stationary-phase and stress promoters as a tool for fine-tuning of expression of recombinant proteins in *Escherichia coli* Journal of Biotechnology 2005 120 25 37 10.1016/j.jbiotec.2005.04.027 16019099

[B23] Sambrook J Molecular cloning: a laboratory manual 2001 CSHL Press

[B24] Srinivasan S Barnard GC Gerngross TU A novel high-cell-density protein expression system based on *Ralstonia eutropha* Applied and Environmental Microbiology 2002 68 5925 5932 10.1128/AEM.68.12.5925-5932.2002 PMC134445 12450812

[B25] Studier FW Protein production by auto-induction in high-density shaking cultures Prot Expr Purif 2005 41 207 234 10.1016/j.pep.2005.01.016 15915565

[B26] Anderson JC Clarke EJ Arkin AP Voigt CA Environmentally controlled invasion of cancer cells by engineered bacteria Journal of Molecular Biology 2006 355 619 627 10.1016/j.jmb.2005.10.076 16330045

[B27] Tsao CY Hooshangi S Wu HC Valdes JJ Bentley WE Autonomous induction of recombinant proteins by minimally rewiring native quorum sensing regulon of E. coli Metab Eng 2010 12 291 297 10.1016/j.ymben.2010.01.002 20060924

[B28] Basu S Mehreja R Thiberge S Chen MT Weiss R Spatiotemporal control of gene expression with pulse-generating networks Proceedings of the National Academy of Sciences of the United States of America 2004 101 6355 6360 10.1073/pnas.0307571101 PMC404049 15096621

[B29] Sayut DJ Niu Y Sun LH Construction and engineering of positive feedback loops Acs Chemical Biology 2006 1 692 696 10.1021/cb6004245 17184133

[B30] You LC Cox RS Weiss R Arnold FH Programmed population control by cell-cell communication and regulated killing Nature 2004 428 868 871 10.1038/nature02491 15064770

[B31] Fuqua C Greenberg EP Listening in on bacteria: Acyl-homoserine lactone signalling Nature Reviews Molecular Cell Biology 2002 3 685 695 10.1038/nrm907 12209128

[B32] Whitehead NA Barnard AML Slater H Simpson NJL Salmond GPC Quorum-sensing in gram-negative bacteria Fems Microbiology Reviews 2001 25 365 404 10.1111/j.1574-6976.2001.tb00583.x 11524130

[B33] Miller MB Bassler BL Quorum sensing in bacteria Annual Review of Microbiology 2001 55 165 199 10.1146/annurev.micro.55.1.165 11544353

[B34] Deana A Ehrlich R Reiss C Synonymous codon selection controls in vivo turnover and amount of mRNA in *Escherichia coli bla *and *ompA *genes Journal of Bacteriology 1996 178 2718 2720 10.1128/jb.178.9.2718-2720.1996 PMC178002 8626345

[B35] Rowe DCD Summers DK The quiescent-cell expression system for protein synthesis in *Escherichia coli* Applied and Environmental Microbiology 1999 65 2710 2715 10.1128/aem.65.6.2710-2715.1999 PMC91400 10347065

[B36] Elledge SJ Davis RW Position and density effects on repression by stationary and mobile DNA-binding proteins Genes & Development 1989 3 185 197 10.1101/gad.3.2.185 2523839

[B37] Eszterhas SK Bouhassira EE Martin DIK Fiering S Transcriptional interference by independently regulated genes occurs in any relative arrangement of the genes and is influenced by chromosomal integration position Molecular and Cellular Biology 2002 22 469 479 10.1128/MCB.22.2.469-479.2002 PMC139736 11756543

[B38] Stevens AM Greenberg EP Quorum sensing in *Vibrio fischeri*: essential elements for activation of the luminescence genes Journal of Bacteriology 1997 179 557 562 10.1128/jb.179.2.557-562.1997 PMC178731 8990313

[B39] Grossman TH Kawasaki ES Punreddy SR Osburne MS Spontaneous cAMP-dependent derepression of gene expression in stationary phase plays a role in recombinant expression instability Gene 1998 209 95 103 10.1016/s0378-1119(98)00020-1 9524234

[B40] Joseph S William RD Molecular cloning: a laboratory manual 2001 1 CSHL Press edition

[B41] Khlebnikov A Datsenko KA Skaug T Wanner BL Keasling JD Homogeneous expression of the P-BAD promoter in *Escherichia coli *by constitutive expression of the low-affinity high-capacity AraE transporter Microbiology-Sgm 2001 147 3241 3247 10.1099/00221287-147-12-3241 11739756

[B42] Khlebnikov A Keasling JD Effect of *lacY *expression on homogeneity of induction from the P-tac and P-trc promoters by natural and synthetic inducers Biotechnology Progress 2002 18 672 674 10.1021/bp010141k 12052093

[B43] Novick A Weiner M Enzyme induction as an all-or-none phenomenon PNAS 1957 43 553 566 10.1073/pnas.43.7.553 PMC528498 16590055

[B44] Dunlap PV Greenberg EP Control of *Vibrio fischeri *luminscence gene expression in *Escherichia coli *by cyclic-AMP and cyclic-AMP receptor protein Journal of Bacteriology 1985 164 45 50 10.1128/jb.164.1.45-50.1985 PMC214208 2995319

[B45] Chaveroche MK Ghigo JM d'Enfert C A rapid method for efficient gene replacement in the filamentous fungus *Aspergillus nidulans* Nucleic Acids Res 2000 28 97 10.1093/nar/28.22.e97 PMC113889 11071951

[B46] Ye R Rehemtulla SN Wong SL Glucitol induction in *Bacillus subtilis *is mediated by a regulatory factor, GutR J Bacteriol 1994 176 3321 3327 10.1128/jb.176.11.3321-3327.1994 PMC205503 8195087

[B47] Richardson SM Wheelan SJ Yarrington RM Boeke JD GeneDesign: rapid, automated design of multikilobase synthetic genes Genome Res 2006 16 550 556 10.1101/gr.4431306 PMC1457031 16481661

[B48] Ames RS Tornetta MA Jones CS Tsui P Isolation of neutralizing anti-C5a monoclonal antibodies from a filamentous phage monovalent Fab display library J Immunol 1994 153 910 8021521

[B49] Rydzanicz R Zhao XS Johnson PE Assembly PCR oligo maker: a tool for designing oligodeoxynucleotides for constructing long DNA molecules for RNA production Nucleic Acids Research 2005 33 W521 W525 10.1093/nar/gki380 PMC1160141 15980526

[B50] DeLisa MP Li JC Rao G Weigand WA Bentley WE Monitoring GFP-operon fusion protein expression during high cell density cultivation of *Escherichia coli *using an on-line optical sensor Biotechnology and Bioengineering 1999 65 54 64 10440671

[B51] Zhang QY Tiersch TR Cooper RK Inducible expression of green fluorescent protein within channel catfish cells by a cecropin gene promoter Gene 1998 216 207 213 10.1016/s0378-1119(98)00272-8 9766968

