# Supplementary figures and images for: The new pLAI (lux regulon based auto-inducible) expression system for recombinant protein production in Escherichia coli
Source: Microb Cell Fact. 2012 Jan 5;11:3. doi: 10.1186/1475-2859-11-3 (PMC3274441; doi:10.1186/1475-2859-11-3)

Additional file 1


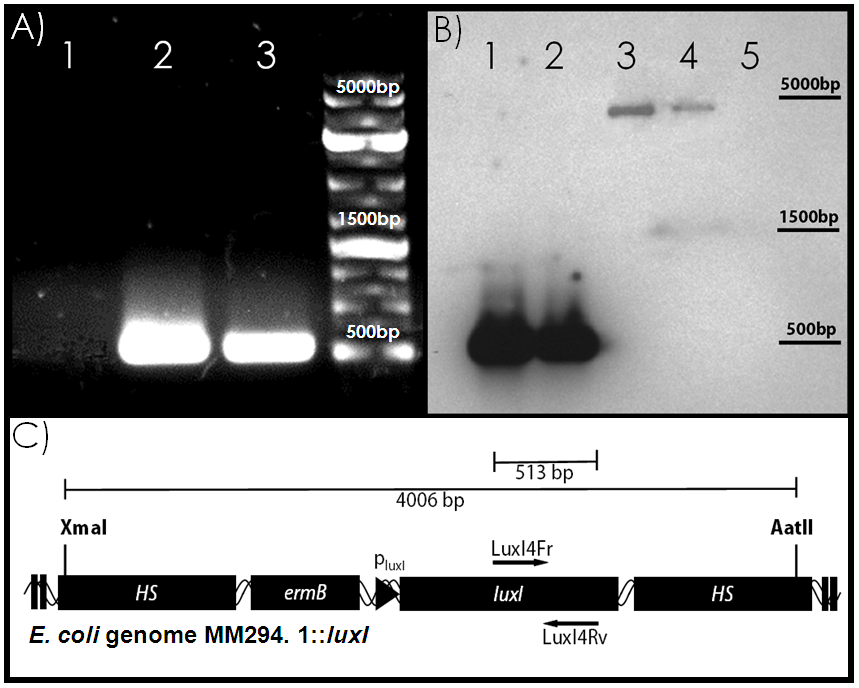


Figure S1


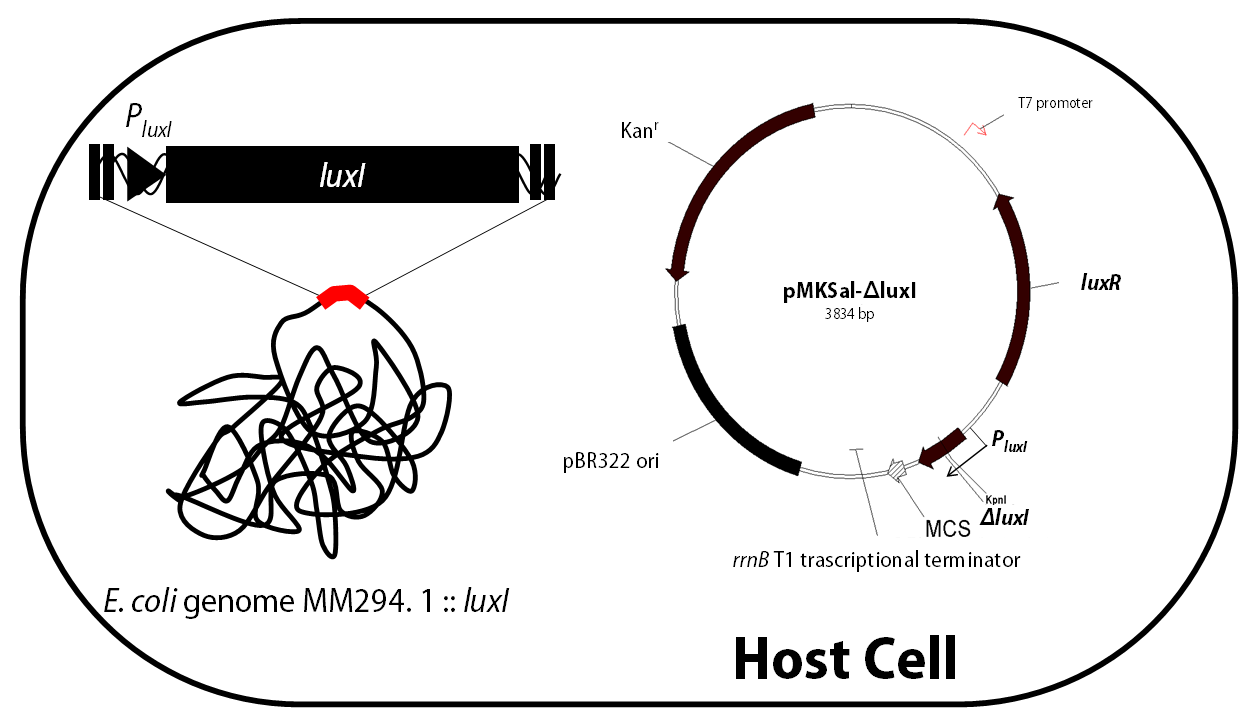


Figure S2


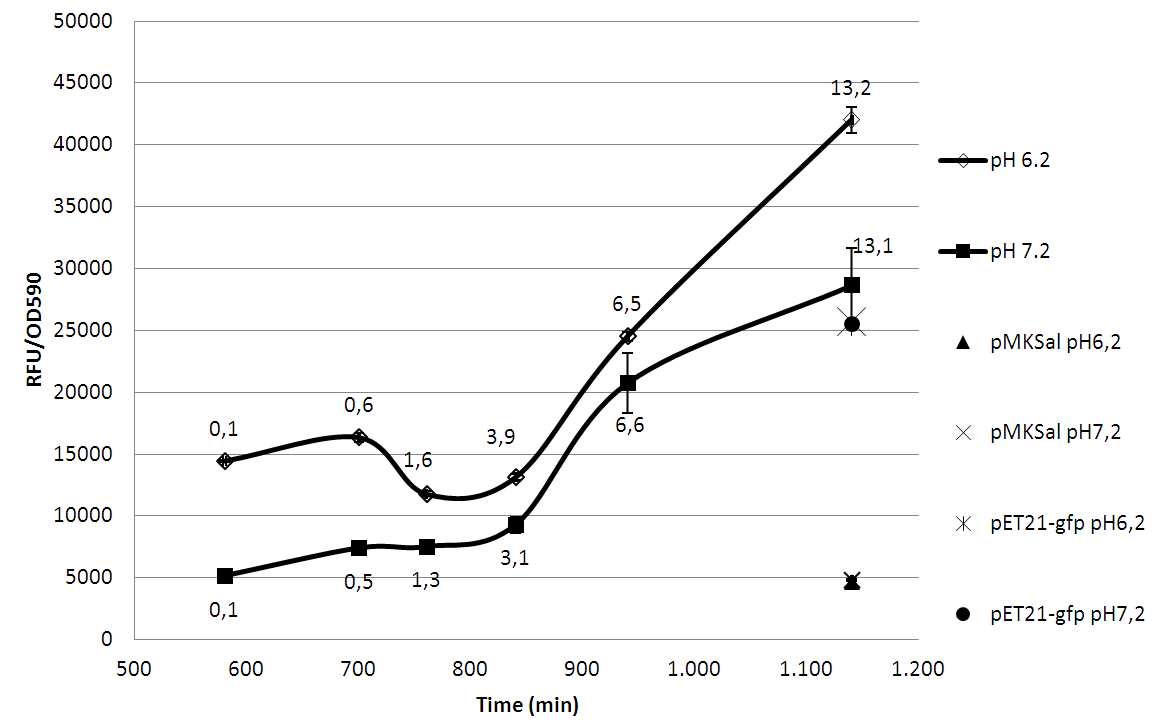


Figure S3


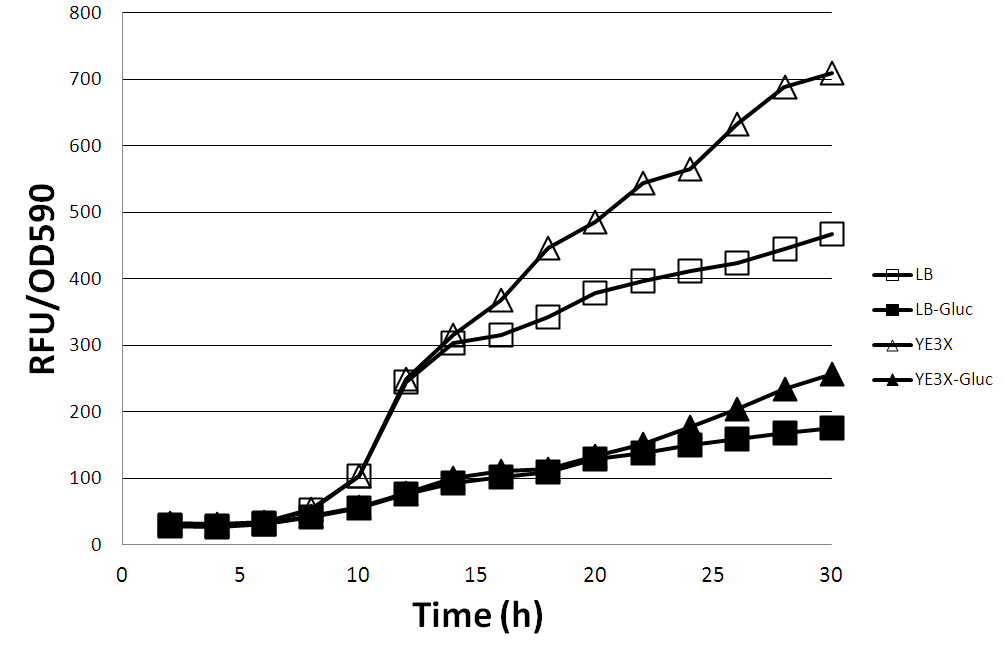


Figure S4


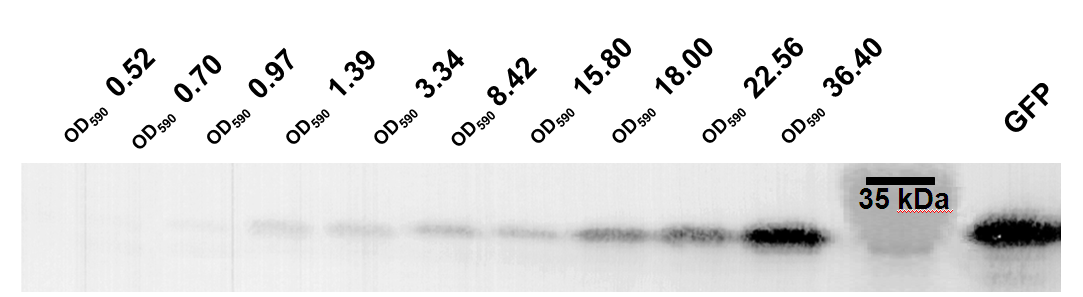


Figure S5

Supplement: Additional file 1 — Figure S1. Molecular characterization of luxI integration into the E. coli genome. A) PCR analysis of MM294.1::luxI. Chromosomal DNA from wild type MM294.1 (line 1), plasmid pGL506 (line 2) and chromosomal DNA of MM294.1::luxI (line 3) were used as a template for PCRs with primers LuxI4Fr/LuxI4Rv, specifically annealing to luxI. A molecular weight ladder is also shown, on the right. B) Southern blot analysis of wild type and MM294.1::luxI mutant strain. Plasmid pGLEM-luxI (line 3; positive control), Chromosomal DNA from MM294.1::luxI mutant (line 4) and wild type MM294.1 (line 5) were digested with XmaI\AatII restriction enzymes. Fragments were separated on an agarose gel and transferred to nitrocellulose membrane for Southern blot analysis using a 513-bp PCR (primers LuxI4Fr/LuxI4Rv) that fully probed within theluxI gene. As a control of the Southern blot efficiency, a 513-bp PCR (primers LuxI4Fr/LuxI4Rv) from plasmid pGL506 (line 1) and chromosomal DNA of MM294.1::luxI (line 2) were loaded onto the agarose gel. C) Schematic representation of luxI integration into the genome. Figure S2. Schematic representation of decoupled luxI/luxR auto-inducible system. LuxI is produced from the genome, reducing the luxI gene dosage to 1/genome, while luxR and the target gene dosages depend on plasmid copy number of pMKSal-ΔluxI. Figure S3. pH effect on the pattern of expression. Comparison of pMKSal-gfp/MM294.1 expression at different cell densities at two different pH set points (6.2 ± 0.1 and 7.2 ± 0.1) controlled during bacterial growth in a bioreactor. Batch processes were carried out in a 7-liter bioreactor (Applikon) under the following conditions: 5L of YE3X containing 15 g/l of glycerol, 25°C, 0.5 VVM (air Volume per Volume of culture medium per Minute) of airflow and a 300-rpm stirrer speed. The glucose was added to the medium where indicated. An Applikon programmable logic controller (ADI1030) was used for maintaining temperature at 25°C and pH set-point. The pH of th [file 1475-2859-11-3-S1.DOCX]
